# Pedestrian Dead Reckoning with Low-Cost Foot-Mounted IMU Sensor

**DOI:** 10.3390/mi13040610

**Published:** 2022-04-13

**Authors:** Shunsei Yamagishi, Lei Jing

**Affiliations:** Graduate School of Computer and Information Systems, The University of Aizu, Aizuwakamatsu 965-0006, Japan; shunsei_research@icloud.com

**Keywords:** PDR, zero velocity update, gait phase estimation, kalman filter

## Abstract

In this paper, we researched Pedestrian Dead Reckoning (PDR) with one foot-mounted IMU sensor. The issues of PDR are magnetism noise and accumulated error due to the noise included in acceleration and gyro data. Two methods are proposed in this paper. First is the gait-phase-estimation method with pitch angle for the Zero Velocity Update algorithm. Second is a method for avoiding accumulated errors by updating the roll and pitch angles with acceleration. The two experiments were conducted to examine the error of gait-phase estimation and distance estimations. The relative error of distance was about 7.40% in the case of walking straight and about 12.27% in the case of a shifting travel direction.

## 1. Introduction

PDR is the one of the technologies to estimate pedestrian position. PDR contributes to navigation systems. Navigation systems should use GPS or PDR technology depending on the situation. For example, a navigation system navigates a person using GPS when they are in the outdoors and navigates a person using PDR when they are in an underground pathway. There are technologies for navigation systems, such as GPS; however, GPS is unsuitable for an indoor navigation system. Navigation systems with IMU sensors are suitable for indoor navigation systems.

IMU-based PDR technologies have been explored in detail by the other papers, and there are advantages, such as no effects from circumjacent objects except for electronic goods in IMU-based PDR. There are technologies to estimate a pedestrian’s position, such as methods using ultrasound waves or beacons. Pedestrian position estimation by these methods are affected by circumjacent objects.

However, there are two main disadvantages in IMU-based PDR methods. First is the accumulated error in estimating velocity. Second is the accumulated error in estimating attitude angles. Generally, the first issue is handled by a Zero Velocity Update algorithm, and the second issue is handled by sensor-fusion methods, such as a Kalman filter and Complementary filter. The development of high accuracy mid-stance detection algorithms for Zero Velocity Updates is needed.

Therefore, in this paper, we proposed new methods to solve these two issues. First is the gait-phase-estimation method for the Zero Velocity Update algorithm. This detects the mid-stance phase. The pitch angle is the focus in this method. When a foot is in mid-stance phase, the pitch angle and the gyro of the foot-mounted IMU sensor will be nearly 0. Secondly, the roll/pitch update method handles the accumulated error in attitude angles. The roll and pitch angles are calculated by integrating the gyro during gait phases, except for mid-stance, and they update the roll and pitch angles using the acceleration measured in each mid-stance phase. The main contributions of this paper are two proposed methods:

- The gait-phase estimation method for Zero Velocity Updates.
- The roll/pitch update method that, during the mid-stance phase, updates the roll and pitch angles using the acceleration measured in each mid-stance phase.

This paper is organized in five sections, with the Introduction in Section 1, Related Works in Section 2, the System and Method in Section 3, Experiments in Section 4 and our Conclusions and Future Work in Section 5.

## 2. Related Works

### 2.1. Application Model

**Figure 1:**
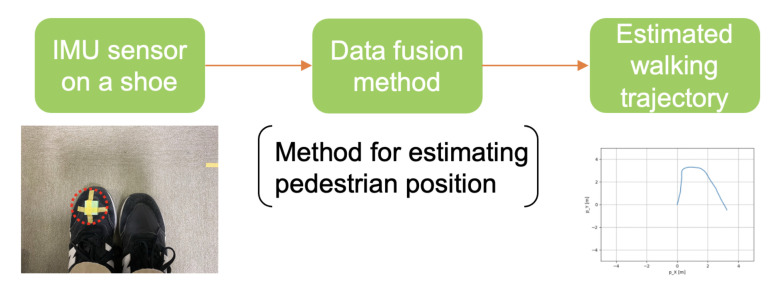
Application model.

A pedestrian wears an IMU sensor on their left shoe. Figure 1 shows application model.

### 2.2. IMU Sensors and Deployment Positions for PDR

In this research, a low-cost IMU sensor was used. There are tactical-grade IMU sensors, such as STIM202 [1], STIM300 [2]. Tactical-grade IMU sensors have much better performance compared with low-cost IMU sensors. However, tactical-grade IMU sensors are not suitable for navigation system in daily life because the price is expensive. In this paper, a low-cost IMU sensor is mounted on a shoe because it is easy to detect the mid-stance phase for Zero Velocity Updates. There are papers on PDR using smartphones, such as [3,4,5]. A paper [4] (A. Poulose et al.) described that the advantage of using smartphone-based position-estimation systems is that this requires no additional peripherals devices except for the smartphone itself. However, according to the paper [3] (S. Park et al.), the zero velocity phase rarely occurs in the case of Zero Velocity Updates (ZUPT) in the smartphone.

### 2.3. Related Works for the First Issue and Second Issue

In the paper [6] (H. Fourati), a method using the Complementary filter instead of Extended Kalman filter was proposed. The paper [6] presented the Quaternion-based complementary filter. MTI-IMU produced by Xsens was used for the experiments of [6]. The measurement error in positioning was about 0.4%.

In the paper [7] (Z. Xiao-dong et al.), a new Zero Velocity Update algorithm using Kalman filter and Particle filter was proposed. An IMU sensor produced by Xsens was used in the experiments of [7]. The measurement error in positioning was less than 0.5%.

In the paper [8] (L.-F.Shi et al.), a novel orientation estimation and gait-phase detection algorithm were proposed. The paper [8] proposed a Zero Velocity Update and orientation and velocity calibration method. The experiments of [8] were conducted indoors and outdoors, attaching an MPU9250 IMMU to the foot. The average distance error was less than 1.2%.

### 2.4. Summary

In previous studies, the orientation and velocity calibration method [8] and methods using sensor-fusion methods, such as Quaternion-based Complementary filter [6], Kalman filter and Particle filter [7] were proposed to handle accumulated error issues. In addition, many gait-phase detection algorithms have been proposed to improve Zero Velocity Updates as well.

Therefore, in this paper, the roll/pitch update method and gait-phase-estimation method are proposed with the aims of handling accumulated errors in attitude angles and improving the Zero Velocity Update method.

## 3. System and Method

### 3.1. System Architecture

**Figure 2:**
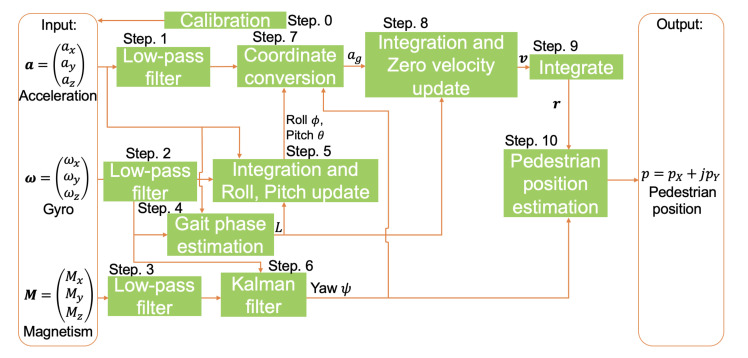
System architecture.

Figure 2 shows the system architecture. There are 11 steps. The gyro and magnetism data are calibrated in step 0. After that, the acceleration, gyro and magnetism data are input. In step 1 to step 3, all input data are filtered by the simple moving average. In step 4, gait-phase estimation is conducted to detect mid-stance phase. In step 5, the gyro data of the pitch angle and roll angles are integrated, and the roll and pitch angles are updated mid-stance using acceleration. In step 6, the gyro and magnetism data are fused to calculate the yaw angle. In step 7, the acceleration is converted from local coordinates to global coordinates. In step 8, the acceleration is integrated to calculate the velocity, and the Zero Velocity Update is conducted. In step 9, the velocity is integrated to calculate the position vector. In step 10, the pedestrian position is estimated using the yaw angle and position vector.

### 3.2. Data Preprocessing (Step 0)

Before the data are processed, the gyro and magnetism data are calibrated. In the gyro calibration, gyro offsets are eliminated by subtracting the gyro data for calibration from the raw gyro data. In the magnetism calibration, the gap of the ellipse center is measured, and the raw magnetism data are modified with the measured gap.

### 3.3. Low-Pass Filter (Step 1, 2, 3)

Acceleration, gyro, and magnetism data are filtered with a low-pass filter and simple moving average. The definition of the simple moving average is shown in Equation (1). $a_{filtered,n}$ and $a_{raw,n}$ denote filtered data and raw data, respectively.

(1) $$a_{filtered,n}=\frac{1}{2w+1}\sum_{k=n-w}^{n+w}a_{raw,k}$$

In this paper, *n* denotes the index. 2w+1 is the window size. The window size is set to 21 (w=10).

### 3.4. Gait Phase Estimation (Step 4)

**Figure 3:**
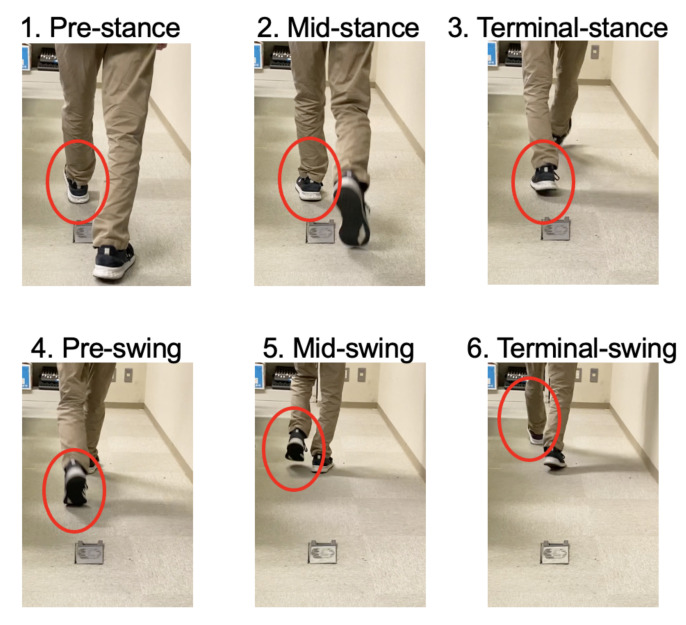
Gait phases (there are six gait phases).

We propose a method to estimate the gait phase with pitch angle. According to the papers [6,9], there are two phases, the stance phase and the swing phase in the gait phase. Furthermore, according to [9], there are the pre-stance, mid-stance and terminal stance in the stance phase, and there are the pre-swing, mid-swing and terminal swing in the swing phase. Figure 3 shows six gait phases. According to the paper [10], the pitch angle is obtained with Equation (2).

(2) $$\theta_{a}=\tan^{-1}\frac{-a_{x}}{\sqrt{a_{y}^{2}+a_{z}^{2}}}$$

Equation (2) is used for our method. Let $\theta_{a,n}=\tan^{-1}\frac{-a_{x,n}}{\sqrt{a_{y,n}^{2}+a_{z,n}^{2}}}$. $\theta_{a,n}$ denotes $\theta_{a}$ measured in *n*-th time. The algorithm to estimate the gait phases is as follows.

Step 1: Conduct the low-pass filter to ${\theta_{a,n}}^{2}$ and ${\omega_{y,n}}^{2}$.

(3) $${\theta_{a,n}^{\prime }}^{2}=\left\{\begin{array}{cc} {\theta_{a,n}}^{2} & \mathrm{if}\;n\leq w\vee N-w\leq n\\ \frac{1}{2w+1}\sum_{i=n-w}^{n+w}{\theta_{a,n}}^{2} & \mathrm{else} \end{array}\right.$$

(4) $${\omega_{y,n}^{\prime }}^{2}=\left\{\begin{array}{cc} {\omega_{y,n}}^{2} & \mathrm{if}\;n\leq w\vee N-w\leq n\\ \frac{1}{2w+1}\sum_{i=n-w}^{n+w}{\omega_{y,n}}^{2} & \mathrm{else} \end{array}\right.$$

N: Length of the data

Step 2: Estimate the gait phase into Label $L_{n}$

(5) $$L_{n}=\left\{\begin{array}{cc} 1 & \mathrm{if}\;\theta_{1}\leq {\theta_{a,n}}^{2}\vee \theta_{2}\leq {\omega_{y,n}}^{2}\\ 0 & \mathrm{else} \end{array}\right.$$

where $\theta_{1}$, $\theta_{2}$ denote thresholds. $\theta_{1}$ is set to 0.1, and $\theta_{2}$ is set to 0.2. If $L_{n}=0$, it is estimated as mid-stance. Otherwise, if $L_{n}=1$, it is not estimated as mid-stance.

### 3.5. Integration and Roll and Pitch Update (Step 5)

The method for avoiding accumulated errors in attitude angles is proposed for use in a ramp. In the paper [8] (L.-F.Shi et al.), they make an assumption that the roll and pitch angles are assumed to be the same as the initial value during each stance phase. In the paper [8], a method based on this assumption is proposed. However, in the case of a ramp, their assumption does not hold.

In the method in this paper, during each mid-stance phase, the roll and pitch angles are updated to $\phi_{a}$ and $\theta_{a}$ as measured in each mid-stance phase. On the other hand, the gyro is integrated during the other gait phases. According to the paper [10], Equation (6) can be used to obtain the pitch angle.

(6) $$\phi_{a}=\tan^{-1}\frac{a_{y}}{a_{z}}$$

Equations (2) and (6) are used for the roll/pitch update method. The trapezoidal rule is used for integration. The trapezoidal rule is shown in the following equation.

(7) $$\int_{a}^{b}f\left(t\right)dt\approx \sum_{n=0}^{N-1}\frac{\Delta t}{2}\left(f_{n}+f_{n+1}\right)$$

Δt is 1/Sampling rate, $f_{0}=f\left(a\right),f_{N}=f\left(b\right)$ and $f_{n+1}=f\left(t+\Delta t\right)$ when $f_{n}=f\left(t\right)$.

The integration and roll and pitch update method is implemented with the recurrence Formula (8).

(8) $$\left[\begin{array}{c} \theta_{n}\\ \phi_{n} \end{array}\right]=\left\{\begin{array}{cc} \left[\begin{array}{c} \tan^{-1}\frac{-a_{x,n}}{\sqrt{a_{y,n}^{2}+a_{z,n}^{2}}}\\ \tan^{-1}\frac{a_{y,n}}{a_{z,n}} \end{array}\right] & \mathrm{if}\;L_{n}=0\\ \left[\begin{array}{c} \theta_{n-1}\\ \phi_{n-1} \end{array}\right]+\frac{\Delta t}{2}\left(\left[\begin{array}{c} \omega_{y,n}\\ \omega_{x,n} \end{array}\right]+\left[\begin{array}{c} \omega_{y,n+1}\\ \omega_{x,n+1} \end{array}\right]\right) & \mathrm{if}\;L_{n}=1 \end{array}\right.$$

**Figure 4:**
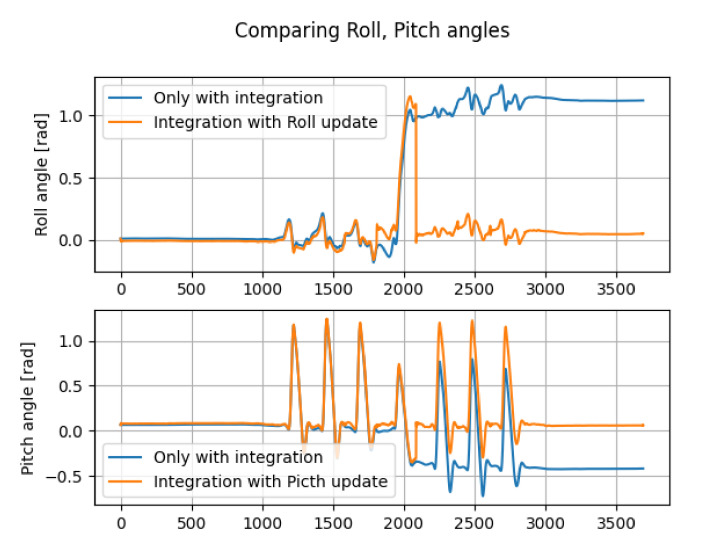
Comparison between attitude angles by only integration and attitude angles by integration with the roll/pitch update (the horizontal axes and vertical axes denote the index and roll or pitch angle [rad], respectively).

Figure 4 shows the comparison between roll and pitch angles obtained by only integration and roll and pitch angles obtained by (8). It is confirmed that the orange lines show less drift than the blue lines.

### 3.6. Kalman Filter (Step 6)

The Kalman filter is implemented with the following status equation and observation equation.

(9) $$\psi_{k}=\psi_{k-1}+\Delta t\omega_{z,k}+w_{k}$$

(10) $$z_{k}=\psi_{k}+v_{k}$$

$w_{k}$, $v_{k}$ and $\psi_{k}$ denote the system noise, the observation noise and the yaw angle in the k−th time, respectively. The observation vector $z_{k}$ is $-atan2\left(m_{x,k},m_{y,k}\right)-\psi_{0}^{\prime }$. $\psi_{0}^{\prime }$ is the average of $-atan2(m_{x,k},m_{y,k})$ from index k=0 to k=399. Let $\psi_{k}$ be status vector ${\hat{x}}_{k}$, A=1, B=Δt, $u_{k}=\omega_{z,k}$ and H=1.

According to [11], the prediction equations are defined from (11) to (12).

(11) $${\hat{x}}_{k}^{-}=A{\hat{x}}_{k-1}+Bu_{k-1}$$

(12) $$P_{k}^{-}=AP_{k-1}A^{T}+Q$$

According to [11], the update equations are defined from (13) to (15).

(13) $$K_{k}=P_{k}^{-}H^{T}{\left(HP_{k}^{-}H^{T}+R\right)}^{-1}$$

(14) $${\hat{x}}_{k-1}={\hat{x}}_{k}^{-}+K_{k}\left(z_{k}-H{\hat{x}}_{k}^{-}\right)$$

(15) $$P_{k}=\left(I-K_{k}H\right)P_{k}^{-}$$

*I* denotes the unit matrix. The filter deviation matrix $P_{0}$ is set to 100, and *Q* and *R* are set to $1.0\times 10^{-5}$, $1.0\times 10^{2}$.

### 3.7. Coordinate Conversion (Step 7)

The acceleration is converted from local coordinates to global coordinates with Quaternion. Quaternion *q* is represented as $q=q_{0}+q_{1}i+q_{2}j+q_{3}k$. Let $u=u_{0}+u_{1}i+u_{2}j+u_{3}k$, $a_{l}=a_{x}i+a_{y}j+a_{z}k$ and $a_{g}=a_{X}i+a_{Y}j+a_{Z}k$. *u*, $a_{l}$ and $a_{g}$ denote Quaternion, which represents rotation, acceleration in the local coordinate and acceleration in the global coordinate, respectively.

(16) $$\left[\begin{array}{c} u_{0}\\ u_{1}\\ u_{2}\\ u_{3} \end{array}\right]=\left[\begin{array}{c} \cos\frac{\psi }{2}\cos\frac{\theta }{2}\cos\frac{\phi }{2}+\sin\frac{\psi }{2}\sin\frac{\theta }{2}\sin\frac{\phi }{2}\\ \cos\frac{\psi }{2}\cos\frac{\theta }{2}\sin\frac{\phi }{2}-\sin\frac{\psi }{2}\sin\frac{\theta }{2}\cos\frac{\phi }{2}\\ \cos\frac{\psi }{2}\sin\frac{\theta }{2}\cos\frac{\phi }{2}+\sin\frac{\psi }{2}\cos\frac{\theta }{2}\sin\frac{\phi }{2}\\ -\cos\frac{\psi }{2}\sin\frac{\theta }{2}\sin\frac{\phi }{2}+\sin\frac{\psi }{2}\cos\frac{\theta }{2}\cos\frac{\phi }{2} \end{array}\right]$$

ψ,θ and ϕ denote the yaw angle, pitch angle and roll angle, respectively. The local coordinate is converted to a global coordinate with the following equation.

(17) $$a_{g}=ua_{l}\bar{u}$$

**Figure 5:**
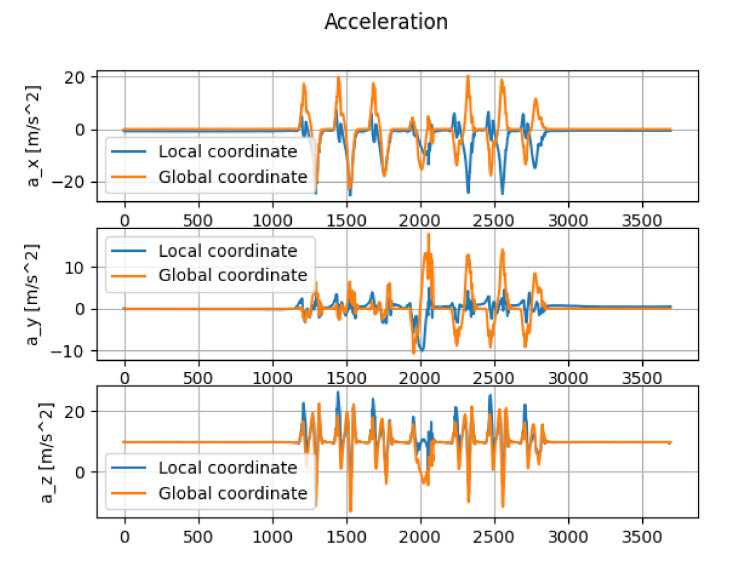
Comparison between local coordinates and global coordinates (The horizontal axes and vertical axes denote the index and acceleration [m/s${}^{2}$], respectively).

$\bar{u}$ denotes the conjugate Quaternion of *u*. Figure 5 shows the comparison between the accelerations in the local coordinate and in the global coordinate.

### 3.8. Integration and Zero Velocity Update (Steps 8 and 9)

Zero Velocity Update is used for reducing the drift. The foot velocity is almost 0 when the foot state is in the mid-stance. From the trapezoidal rule and Zero Velocity Update, the foot velocity $v_{n}$ and position vector $r_{n}$ are calculated with the following recurrence equation.

(18) $$v_{n}=\left\{\begin{array}{cc} 0\in R^{2} & \mathrm{if}\;L_{n}=0\\ v_{n-1}+\frac{\Delta t}{2}\left(\left[\begin{array}{c} a_{X,n}\\ a_{Y,n} \end{array}\right]+\left[\begin{array}{c} a_{X,n+1}\\ a_{Y,n+1} \end{array}\right]\right) & \mathrm{if}\;L_{n}=1 \end{array}\right.$$

(19) $$r_{n}=r_{n-1}+\frac{\Delta t}{2}\left(\left[\begin{array}{c} v_{X,n}\\ v_{Y,n} \end{array}\right]+\left[\begin{array}{c} v_{X,n+1}\\ v_{Y,n+1} \end{array}\right]\right)$$

$$\left(v_{0}=0\in R^{2},r_{0}=0\in R^{2}\right)$$

**Figure 6:**
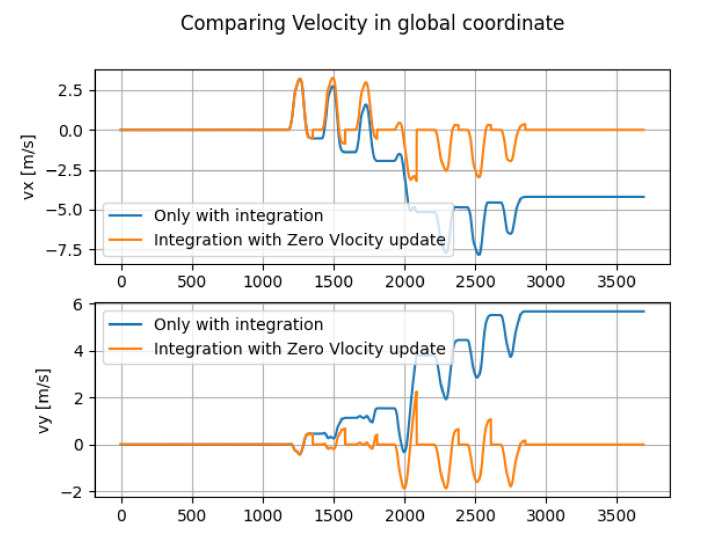
Comparison between the velocity obtained by only integration and the velocity obtained by the Zero Velocity Update and integration (the horizontal axes and vertical axes denote the index and velocity [m/s], respectively).

Figure 6 shows the comparison between the velocity obtained by only integration and the velocity obtained by the Zero Velocity Update and integration.

### 3.9. Pedestrian Position Estimation (Step 10)

**Figure 7:**
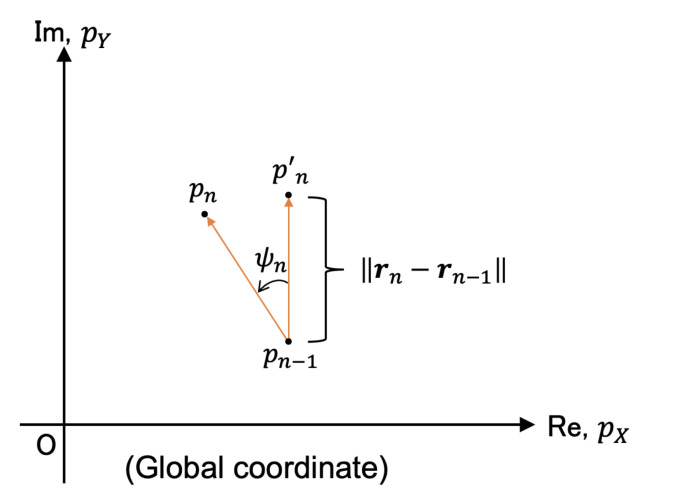
The pedestrian position in the global coordinates ($p_{n}\;\mathrm{and}\;p_{n-1}$ denote the current pedestrian position and previous pedestrian position, respectively. Coordinate $\left(p_{X},p_{Y}\right)$ is the global coordinate of the floor).

The pedestrian position is estimated on the complex plane using the following equations (Figure 7).

(20) $$p_{n}^{\prime }=p_{n-1}+\left|\right|r_{n}-r_{n-1}\left|\right|i$$

(21) $$p_{n}=\left(p_{n}^{\prime }-p_{n-1}e^{i\psi_{n}}\right)+p_{n-1}$$

$$\left(p_{0}=0\right)$$

$p_{n}$ is the pedestrian position.

## 4. Experiments

**Figure 8:**
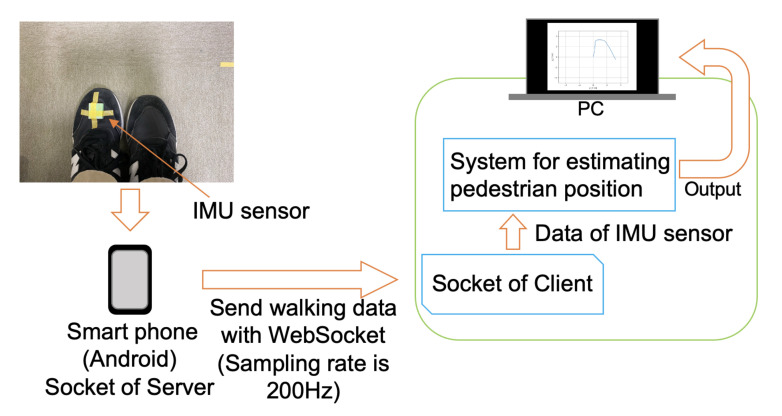
Implementation of the experiments.

**Figure 9:**
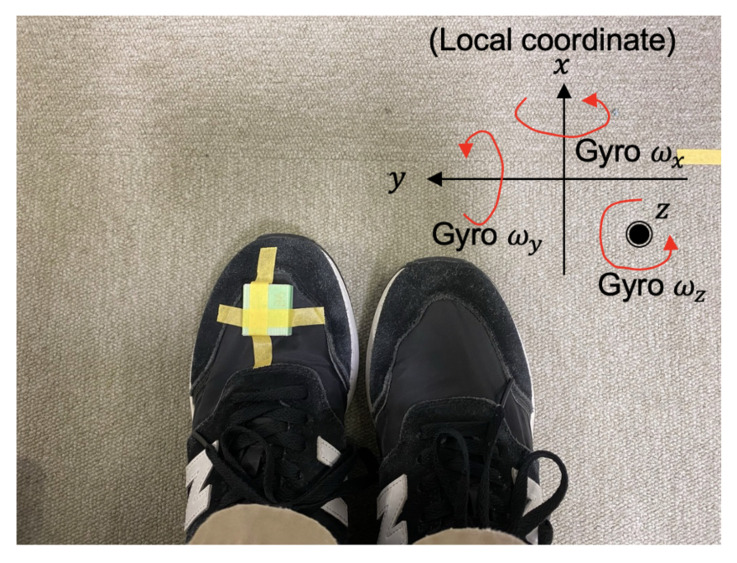
The placement location of the IMU sensor on a shoe.

The two experiments were conducted for accuracy examination of the system. The experiments were conducted indoors. Figure 8 shows the implementation of the system, and Figure 9 shows the location of the IMU sensor. The IMU sensor (Wonder-Sense, developed in our laboratory) was used.

### 4.1. Exp. 1: Gait Phase Estimation Test

This experiment’s objective is accuracy examination for gait-phase estimation. We defined the True label by synchronizing the collected data with videos taken during collecting the data. If the True label is 0, this indicates mid-stance. On the other hand, if the True label is 1, this indicates the other gait phases. The estimation error (ER) is calculated with the below equation.

(22) $$\mathrm{ER}\;=(1-\frac{\mathrm{True}\;\mathrm{Positive}}{\mathrm{True}\;\mathrm{Positive}+\mathrm{False}\;\mathrm{Negative}})\times 100\;[\%]$$

**Figure 10:**
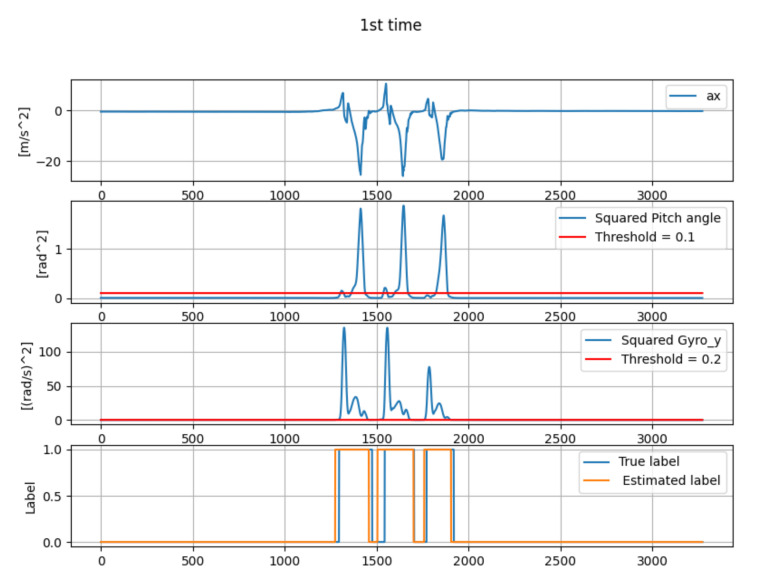
The wave-form of the data from the first data collection.

**Figure 11:**
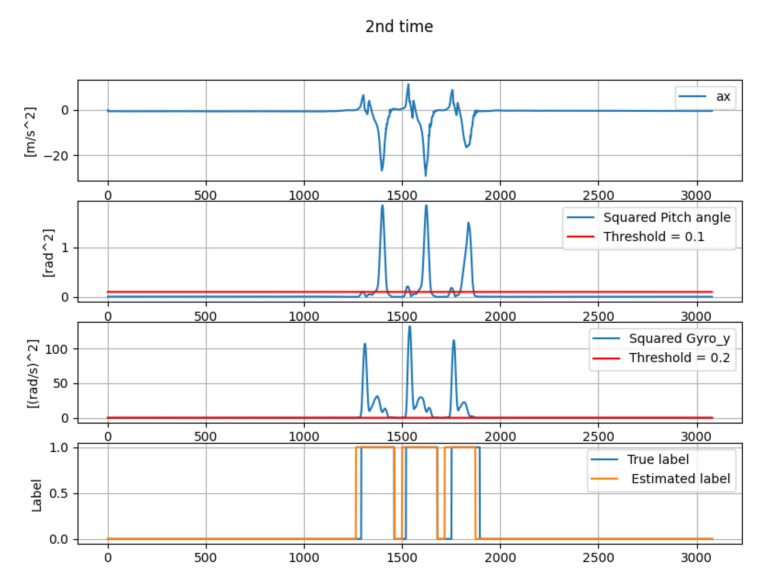
The wave-form of the data from the second data collection.

**Figure 12:**
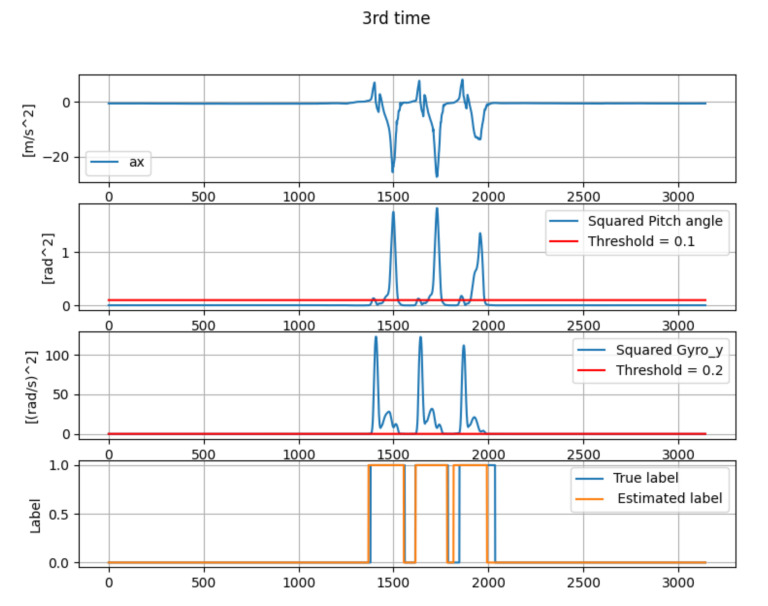
The wave-form of the data from the third data collection.

Table 1 shows the estimation error of mid-stance by the gait-phase-estimation algorithm shown in Section 3.4. Three sets of straight walking data of one person were collected. Figure 10, Figure 11 and Figure 12 show the wave-form of data collected in first time, second time and third time. The wave-form in the first line shows the acceleration data in the *x* axis (acceleration data of the traveling direction) in local coordinates. The wave-form in the second line shows the squared pitch angle ${\theta_{a,n}^{\prime }}^{2}$. The wave-form in the third line shows the squared pitch angle’s gyro ${\omega_{y,n}^{\prime }}^{2}$. The wave-form in the fourth line shows a comparison between the true label and estimated label.

The gait-phase-estimation algorithm should be improved from the data in Table 1. Estimation error of mid-stance was about 7.34% in average. However, each estimation error is scattered. A reliable gait-phase-estimation algorithm should have a lower error and low variance.

### 4.2. Exp. 2: Test for Estimation of Walking Trajectory

This experiment’s objective was the examination of the accuracy of the trajectory estimation. The relative error (RE) shown in the following equation calculates the accuracy of the distance estimation.

(23) $$\mathrm{RE}=\frac{\mathrm{Estimated}\;\mathrm{distance}-\mathrm{Walked}\;\mathrm{distance}}{\mathrm{Walked}\;\mathrm{distance}}\times 100\;[\%]$$

**Figure 13:**
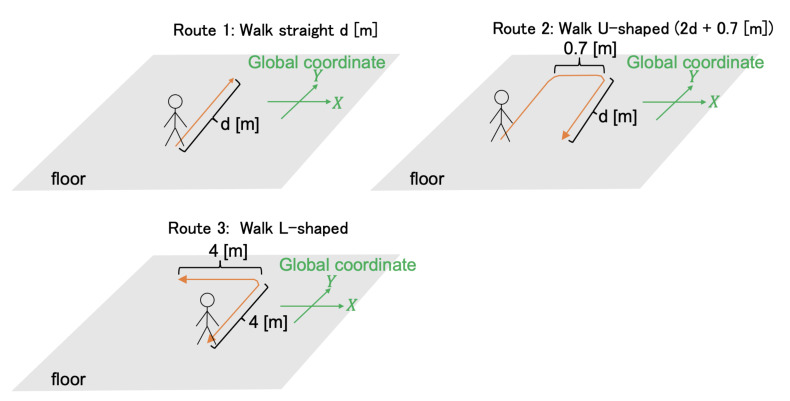
The walking routes of Experiment 2.

**Figure 14:**
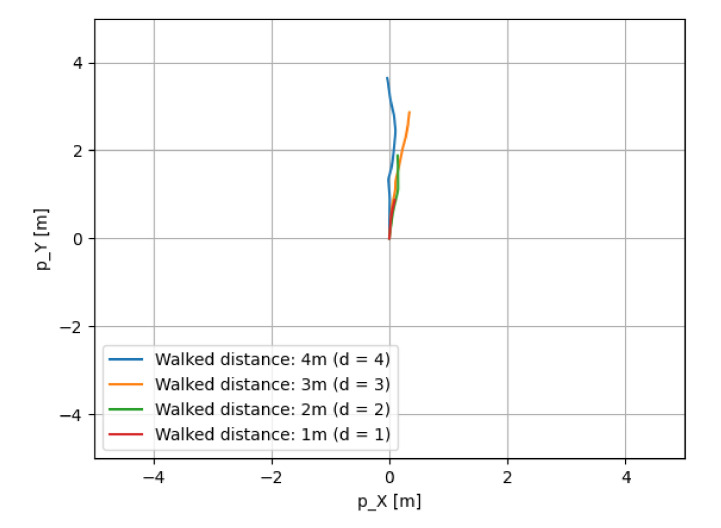
The results of route 1.

**Figure 15:**
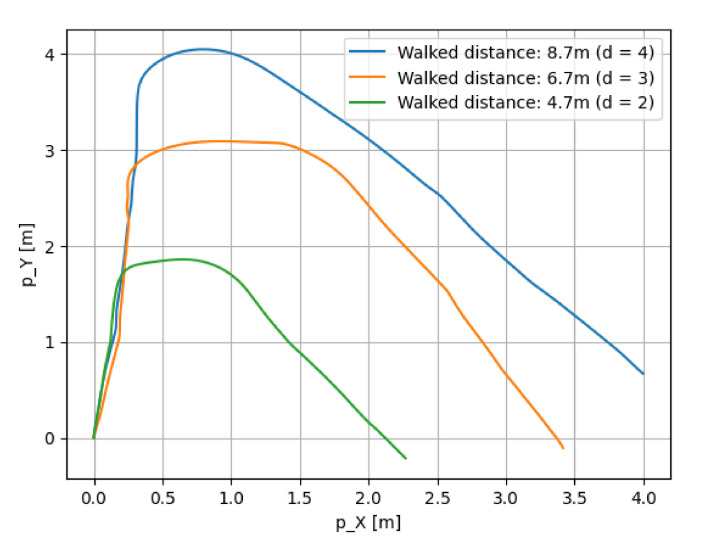
The results of route 2.

**Figure 16:**
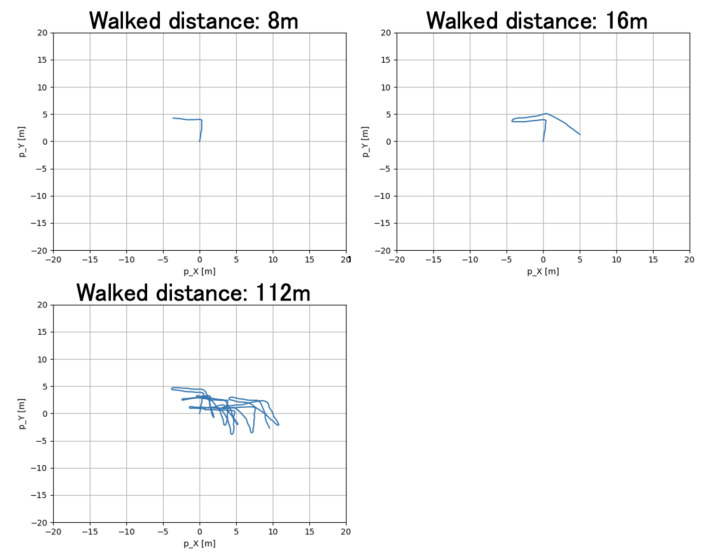
The results of route 3.

Figure 13 shows the walking routes of this experiment. Figure 14, Figure 15 and Figure 16 show the estimated walking trajectory. Table 2, Table 3 and Table 4 show the results of the estimated distance. The walking data of one person were collected. This experiment was conducted in the laboratory.

The relative error of distance is much larger than in related studies, and the yaw angle estimation is not good as shown Figure 15 and Figure 16. One of the possible reasons for the bad yaw angle estimation is the effect of magnetism from the other electronic devices. It is possible that the Kalman filter could not accurately estimate the yaw angle due to the large magnetism noise. Therefore, the yaw angle estimation in this paper should be improved referring to papers on the heading estimation method, such as [12,13].

## 5. Conclusions and Future Work

The norm of the relative errors of distance was about 7.40% in the case of straight walking and a short distance. On the other hand, the relative error of distance was about 12.27% in the case of a shifting travel direction. In comparison with with related research, the relative errors of the system in this paper were larger. In particular, the relative error of distance was much larger in the shifting travel direction. Future work must verify the accuracy of the system for the case of walking on a ramp. We could not verify this due to time constraints in our research. In addition, the roll/pitch update method in Section 3.5 was proposed in the case of a ramp, and the gait-phase-estimation method in Section 3.4 was proposed in the case of no ramp. Therefore, the gait-phase-estimation method should be modified when the roll/pitch update is verified in the case of walking on a ramp.

## Figures and Tables

**Table 1:** The results of Experiment 1.

	Estimation Error of Mid-Stance [%]	Measurement Time [s]
first time	7.38	16.2
second time	4.66	15.3
third time	9.99	15.6

**Table 2:** The results of route 1.

Walked Distance [m]	Estimated Distance [m]	Relative Error [%]	Measurement Time [s]
1 (d = 1)	0.88	−11.964	11.9
2 (d = 2)	1.893	−5.355	13.3
3 (d = 3)	2.887	−3.752	14.3
4 (d = 4)	3.658	−8.545	12.5

**Table 3:** The results of route 2.

Walked Distance [m]	Estimated Distance [m]	Relative Error [%]	Measurement Time [s]
4.7 (d = 2)	4.911	4.491	17.0
6.7 (d = 3)	7.846	17.103	17.6
8.7 (d = 4)	9.061	4.147	18.3

**Table 4:** The results of route 3.

Walked Distance [m]	Estimated Distance [m]	Relative Error [%]	Measurement Time [s]
8	7.883	−1.466	20.8
16 (1 round-trip)	19.688	23.049	28.9
32 (2 round-trip)	37.357	16.739	47.5
48 (3 round-trip)	54.02	12.541	67.8
64 (4 round-trip)	71.809	12.201	85.8
80 (5 round-trip)	91.037	13.797	101.4
96 (6 round-trip)	110.206	14.798	114.1
112 (7 round-trip)	128.723	14.931	129.9
